# Supplementary material for: Period poverty: The perceptions and experiences of impoverished women living in an inner-city area of Northwest England
Source: PLoS One. 2022 Jul 14;17(7):e0269341. doi: 10.1371/journal.pone.0269341 (PMC9282460; doi:10.1371/journal.pone.0269341)
Supplement: S1 File — (PDF) [file pone.0269341.s001.pdf]

GUIDE – FOCUS GROUP DISCUSSION – PERIOD STUDY - WOMEN

MODERATOR: Document required information as appropriate for each FGD using the formats provided below. Date: ____/______/_______

Initials: Moderator: ______ Note Taker______ Recorder Number: ____ Folder/File Name (location on recorder): ____________________

Interview location (Venue): ______________________________________ FGD Group: _______________________________ FGD Number: _____

Time Start: _____________ Time stop: ______________ No. Participants at start of FGD: _______ No. Participants at the end of FGD: ________

Demographic information for every FGD participant *[to be completed on a one-to-one basis, immediately after consent is obtained]*

**Participant number or Fake name**	**Age in years**
1	
2	
3	
4	
5	
6	
7	
8	
9	
10	
11	
12	

COMMENTS – reasons for withdrawal, refusal, ambience of FG, level of interest, disagreements, etc

Introduction

Thank you for your willingness to take part in this group discussion. My name is **[Name]**. I am from the Liverpool School.
We are interested in hearing from you about menstrual needs and practices among women who visit this supported accommodation, or foodbank, to understand the problems and solutions faced on dealing with periods

Focus groups like this are informal; you can talk about anything you think is important for us to know. I also want to remind you that everything we talk about today is confidential. No one will hear this tape except for people working on the project. Whenever we write a report, we will use numbers or fake names so no one can identify you. If there are any questions you'd rather not answer, just let me know - that's fine.

Your frank responses and discussion will be most helpful to us as we try to clarify the critical issues and consider what strategies can be developed to help resolve problems. Your answers are based on your experiences, observations, or feelings, and will not be considered "right" or "wrong", because we want to know what you think. Everyone's views are equally important. It's fine to disagree with other people's views, but if you do, it's important to disagree in a respectful and polite manner. It's important for you to talk in turns to speak, because if you all speak at once, we will not have a clear recording. If you disagree with something anyone says, you can say 'I disagree' and then wait for them to finish before you speak.

- **Explain the role of note-takers and tape-recorder**
- **Give a few minutes for answering any questions regarding the FGD**

Please note the questions here:

Main questions	Further probing on details
Can you tell us what menstruation means to you	Does anyone have anything positive / negative to say about menstruation?<br><br>What is the best thing about menstruation?<br><br>What is the worst thing about menstruation? (Prompts: do you agree / disagree – why?)
Have your views always felt like this or have they changed?	for anyone whose opinions /experiences have changed – why was this?
Can you tell us what menstrual products/materials are available in your area?<br><br>Are there any other products that you have heard of – but might not have seen or are available to you?	for each they list, explore what they think of them- would they choose to use if they could–<br><br>if not why not – discuss reasons for this, e.g. expense / availability / quality /practicality /culture
Are there any restrictions that menstruation places on life for you/your friends, others here?	What are these? Why do they happen?<br><br>probe - Is this related to having to budget / not having access to product/materials<br><br>Probe on leakage / culture / washing and drying / changing / disposal
What do you think about the affordability of menstrual products/materials available?	Are they affordable –<br><br>How do you/your friends / others in similar circumstances afford, or do you have to go without / improvise?<br><br>How important is it to you and your friends to have such products/materials?<br><br>what do you/friends have to do to get them?

	how else do you/your friends deal with affording to access these (e.g. wear one pad for much longer) – what are consequences?<br><br>Please tell us more about this and how it could feel……
If you / your friends / others here get hold of them somehow?	If so – how? How easy is this?<br><br>Do you / they 'borrow'/ Do you/they have to get these from foodbanks Do you get them on behalf of others? If so, who do you get them for? (prompt) - are they available / are there enough, are they free from the foodbank, should they be?<br><br>Do you/they have to budget or find ways to get money to be able to get products/materials?<br><br>To pay for products/materials, are there things you / they have to go without – what are these? How do you decide what to go without?<br><br>Do you / they have to barter or exchange things to get hold of any products/materials? How do you/they do this?<br><br>have you ever heard if some women/girls coerced by men to give sex for menstrual needs? – any examples (without naming)
If you can't / don't purchase or get hold of products/materials - Do you / your friends have to improvise?	how / in what way? - what is the consequence<br><br>probe on stigma, shame, smelling, sores, discomfort; infections
Do you know of any women who resort to quite unusual /drastic measures to obtain menstrual products/materials such as shoplifting or getting paid for sexual favours?	Can you tell us more?<br><br>Do you think this sort of thing happens often / to many women?<br><br>What are the consequences that happen because of this?

	probe – humiliation, shame, police, health impacts
Do you / your friends / others here have any difficulties changing your menstrual product/materials (if applicable).	Is it easy to find places to change /dispose of soiled-used materials?<br><br>what places? Are they suitable places? What about at night?
Do you / your friends / others here have any difficulties being able to wash and dry yourself and materials used during menstruation?	Is it easy to find places to wash yourselves/ wash and dry materials e.g. cloth?<br><br>what places? Are they suitable places? What about at night?
What about period (menstrual) pain – Do you / your friends / others here need help managing menstrual pain?	Are you /your friends / others here able to get hold of anything that helps?<br><br>(for yes): What is this? How do you / they manage to get hold of these things?<br><br>(for no): What would you / your friends like to have? Is there anything you / your friends can do to make yourself feel better?<br><br>What is menstrual pain like for you / your friends / others here?<br><br>Does it place any restriction on what you / your friends / others here can do, or the way you/they behave?
What changes could be made to make menstruation a better experience for women	Product/material related / cost / availability / washing / changing / disposal policies
If there was just one thing that could be introduced to improve menstruation for women	what would this be? (note - see if FGD members can come to agreement)
