# Supplementary material for: Period poverty: The perceptions and experiences of impoverished women living in an inner-city area of Northwest England
Source: PLoS One. 2022 Jul 14;17(7):e0269341. doi: 10.1371/journal.pone.0269341 (PMC9282460; doi:10.1371/journal.pone.0269341)
Supplement: S2 File — (PDF) [file pone.0269341.s002.pdf]

INDEPTH INTERVIEW GUIDE – PERIOD STUDY - WOMEN

INTERVIEWER: Document required information as appropriate for each IDI using the formats provided below. Date: ____/______/_______

Initials: Interviewer: ______ Recorder Number: ____ Folder/File Name (location on recorder): ____________________

Interview location (Venue): ________________________________ Participant code: ____________________ Participant age: __________

Current lodging: ____ Time Start: ____________ Time stop: ____________

COMMENTS – reasons for withdrawal, refusal, ambience of interview, level of interest, etc

Introduction

Thank you so much for your willingness to take part in this interview. My name is **[Name]**. I am from the Liverpool School of Tropical Medicine. We are interested in hearing from you about menstrual needs and practices among women who visit this support accommodation, or foodbank, to understand the problems and solutions faced on dealing with periods.

This interview will be informal; you can talk about anything you think is important for us to know. I also want to remind you that everything we talk about today is confidential. No one will hear this tape except for people working on the project. Whenever we write a report, we will use numbers or fake names so no one can identify you. If there are any questions you'd rather not answer, just let me know - that's fine.

Your frank responses and discussion will be most helpful to us as we try to clarify the critical issues and consider what strategies can be developed to help resolve problems. Your answers are based on your experiences, observations, or feelings, and will not be considered "right" or "wrong", because we want to know what you think. Everyone's views are equally important.

- **Explain the need for tape-recorder**
- **Give a few minutes for answering any questions regarding the interview**

Please note the questions here:

Main questions	Further probing on details
Can you start by telling me a little bit about yourself and your background?<br><br>Can you tell me about your current living accommodation	How old are you? Where do you come from? Do you have family? (**probe on anything relevant to get them talking and opening up a little**)<br><br>where do you live, how long have you been there? are you living here on your own or with anyone else?
Can you tell me what menstruation means to you?<br><br>Have you always felt like this?	Do you think there is there anything positive / negative about menstruation?<br><br>If yes: where did you form your early opinion / what or who were your influences?<br><br>If no: What was your earlier opinion? Why have you changed your opinion? What or who are your current influences?
Can you tell me what having a period is physically like for you at the moment…?	how regular they are, for example do you have them every month? How long do they last? Are they light or heavy?
Moving away from talking about your period itself, can you tell me how you feel in the day or so leading up to your period	Do you think about it? (what do you think / why?)<br><br>Do you plan for it? (how? Why? What happens if you don't plan?)<br><br>Do you try and budget for it? (how, what do you do?)<br><br>When it starts what happens? How do you feel? (physically / emotionally)
Do you suffer from period pain?	If yes: How bad is the pain? Does it restrict you from doing anything (what / how?) Are you able to take anything to help you?<br><br>If yes: what? How much does it help? How do you get hold of it? Can you get this every month you need? If no: why is this?

Do you usually feel unwell, (for example dizzy or very tired) during your period?	If yes: Do you ever need to rest – Do you have anywhere to go, what do you do?
What do you think about the affordability of menstrual products/materials available?<br><br>Can you tell me what menstrual products have you heard of?	Are they affordable?<br><br>How important is it to you to have such products/materials?<br><br>What product would you prefer to use if you had a choice of any? What are the reasons for your choice?
Can you tell me about the products/materials you usually use when you are having your period……?<br><br>What do you usually use?<br><br>How comfortable do you find (these materials / products)?	**If manufactured (disposable pads / tampons /cup etc):**<br><br>Can you usually get hold of these? How do you acquire them? (Do you buy them or get given or..?)<br><br>Is this usually from the same source or do you acquire from different sources / ways at different times?<br><br>Do you ever get products/materials from a foodbank? Do you ever get these for other members of your family or friends etc? Probe: Who do you do this for? / how often do you help them by doing this? Does this ever cause problems?<br><br>If you have pads/tampons sometimes, do you have enough to last for your whole period? If not, why - what happens?<br><br>How often do you need to use impromptu materials? how do you feel about this? How do you get hold of these materials?<br><br>**If impromptu / improvised materials: (paper/cloth/cardboard etc):**<br><br>How do you feel about using these materials? How do you get hold of these?<br><br>**For all**: Do you ever get sore / chafing – is this often / occasionally?

	Is there a specific situation when this occurs eg if you wear for too long, or use a certain material?<br><br>What do you do to help prevent this from happening? Is there anything you can do / use to provide relief?<br><br>Have you ever had to resort to anything that added extra problems for you- such as stealing / shoplifting / performing favours or exchange sex for money to obtain materials or products; or money to buy products<br><br>Can you tell me more about this. Was this a one off or does this happen regularly? How do you feel about this?
How reliable do you find these products / materials	Do you ever / often leak? Do you worry about this? Is leakage usually minimal or is this a lot? How does this make you feel?<br><br>Do you ever have to dispose of any clothing because you have leaked? If yes: does this happen often, do you need to replace your clothing? is this expensive for you?
Can you tell me what happens when you need to change your (product / material)?<br><br>Where / what sort of place do you usually change your product / material?	How long do you usually last before you have to change it? Is this about the right length of time for you or do you ever have to wait before you change / why?<br><br>Is this a good place to change? Why / not?<br><br>Have you tried (unsuccessfully) other places to change – tell us about this.<br><br>What happens at night – can you change / where? are there any hazards to doing this?
Can you tell me about how you usually wash yourself during menstruation?	Where / how do you do this? Are there any problems or is this ok? (If any problem), please tell me more about this.

What about washing your clothes, and any materials / products used for your menstruation?	Where / how do you do this? Are there any problems or is this ok? (If any problem), please tell me more about this – how do you dry reusable material / clothing, are there problems or repercussions of doing this?<br><br>If you wash items, can you easily put them somewhere to dry? Are there any problems that arise in trying to dry them? Do you ever wear these when still damp? Does this cause any issues, please explain?
Can you tell me about disposing of any soiled products / materials?	How do you deal with this? Does it vary by material? Or where you are?<br><br>Do any problems arise in trying to dispose of any items (if yes: what are these / how do you try and deal with this?
Does having or period restrict your life in any way?<br><br><br>Would you ever consider using something like the coil – which stops you from having a period (but is not permanent)	If yes: Can you tell me how and why this happens? How does this make you feel?<br><br>Are there any consequences from this restriction (if yes: what?)<br><br>If yes: no: don't know – probe why, what are their thoughts on this
Is there anything you would like to see changed in relation to helping women with menstruation?<br><br>Is there anything important in relation to menstruation that we have not covered that you want to mention?	Probe: physically, emotionally, washing / changing, restrictions
