# Supplementary material for: Period poverty: The perceptions and experiences of impoverished women living in an inner-city area of Northwest England
Source: PLoS One. 2022 Jul 14;17(7):e0269341. doi: 10.1371/journal.pone.0269341 (PMC9282460; doi:10.1371/journal.pone.0269341)
Supplement: S3 File — (PDF) [file pone.0269341.s003.pdf]

INDEPTH INTERVIEW GUIDE – PERIOD POVERTY - STAFF

INTERVIEWER: Document required information as appropriate for each IDI using the formats provided below. Date: ____/______/_______

Initials: Interviewer: _____ Recorder Number: ___ Folder/File Name (location on recorder): ___________________

Interview location (Venue): ____ Participant code: ____________________ Participant age: _______

Time Start: ____________ Time stop: _____________

COMMENTS – reasons for withdrawal, refusal, ambience of interview, level of interest, etc

Introduction

Thank you so much for your willingness to take part in this interview. My name is **[Name]**. I am from the Liverpool School of Tropical Medicine. We are doing a research study on Menstrual Needs of Women in Liverpool.

We're interested in hearing from you about menstrual practices among women who face deprivation in Liverpool, understanding the problems and solutions faced, and particularly to better understand among women facing difficult day-to-day challenges. This interview will be informal; you can talk about anything you think is important for us to know. I also want to remind you that everything we talk about today is confidential. No one will hear this tape except for people working on the project. Whenever we write a report, we will use numbers or fake names so no one can identify you. If there are any questions you'd rather not answer, just let me know - that's fine.

Your frank responses will be most helpful to us as we try to clarify the critical issues and consider what strategies can be developed to help resolve problems. Remember, your answers to our questions will not be considered "right" or "wrong", because we want to know about what people think. They are merely information you will provide based on your experiences, observations, or feelings.

- **Explain the need for tape-recorder**
- **Give a few minutes for answering any questions regarding the interview**

Please note the questions here:

**Main questions**	**Further probing on details**
Can we start off with you telling me a little bit about the organisation that you work for.	What services does it provide / what does it do?<br><br>What does your job involve / how long have you worked here
What women use your organisation?<br><br>What are the typical circumstances which occur that mean women need to utilise these services (e.g. ~~housing shelter~~support accommodation / food bank?<br><br>Is this service open to all women, or does it select based on defined criteria or circumstances?<br><br>Are any women turned away or restricted?	Age range / ethnicity / marital status….?<br><br>What criteria is used in determining who are selected?<br><br>If yes: does this happen often? Are some women refused help, or restricted to only a few services?<br><br>Probe – who; probe on what services are restricted, what remain available
Does your service provide menstrual hygiene products to women?	If yes:<br><br>How long have you been providing these to women? What products do you provide? Are these what women want or prefer? (what would they prefer / why? Is there a reason you do not / cannot provide them?)<br><br>how does your organisation obtain these products? Do you have a regular supply / regular supplier/s?<br><br>Do you have enough supplies for the requirements of these women? What happens if there are not enough supplies to meet the demand? (do you have to restrict the number of pads / tampons that you give out, or is it first come, first

	served etc? Do you know how the women manage if this happens? what do they use / do they have to budget/ do they have to improvise - what do they use? If so: do you know of any consequences of this?)<br><br>If yes – are they provided free of charge or does it incur some costs for the women? Please explain what these are – is it based on specific criteria (e.g. free for some, costs for others)<br><br>If no:<br><br>do you think they should? Is there demand for them? Do you know how women who use the (support accommodation / foodbank) manage their menstrual needs? (what do they use / do they have to budget/ do they have to improvise - what do they use? If so: do you know of any consequences of this?<br><br>Do you know of any women who resort to quite unusual /drastic measures to obtain menstrual products/materials such as shoplifting or getting paid for sexual favours? (Can you tell us more? / do you think this happens often or to many women? / are you aware of any consequences because of this - humiliation, shame, police, health impacts)
Foodbank specific service	Does the foodbank run out of materials, if yes is this at particular/regular times; can this be resolved?<br><br>Does the foodbank have a quota for menstrual product supplies, relating to anticipated number of women in need? Is the quota for any menstrual product or is this split per type of product? (e.g a set number of proportion of pads versus tampons) What about flow related? (e.g. a set number / proportion of heavy and light flow supplies)

	Would these supplies adapt to alternative types of products not yet available e.g. menstrual cups, or period panties?<br><br>What would be required to help develop policy to organise this? (probe – what data is needed, that research can contribute towards)<br><br>Kindly let us know any other aspects relating to menstruation and your foodbank facilities that we may have overlooked.
Do women talk about their menstrual needs / issues /experiences with staff / other women at the support accommodation / food-bank?	What sort of things do they say? Can you identify any particular issues that affect these women?<br><br>What about issues with menstrual pain?<br><br>What about issues cleaning themselves?<br><br>What about cleaning / drying their clothes and / or menstrual cloth / rags etc
Accommodation-specific questions	What are facilities in the support accommodation for women to wash, change, privacy to clean themselves and dry soiled materials; availability of soap etc<br><br>Do some women requesting services stop by to manage their menstruation here without sleeping overnight?<br><br>Are certain menstrual supplies often unavailable from the supplies. If yes, what are most prominent?<br><br>If yes, what would be required to help develop policy to organise this? (probe – what data is needed, that research can contribute towards)<br><br>Kindly let us know any other aspects relating to menstruation and your support accommodation facilities that we may have overlooked.

Are you aware how much of an issue menstruation and managing it, is to these women's lives?	Can you tell me more about this?
Do you think there is anything else your service could do, or could do better, to help your clients with their menstrual needs?	If so: What would this involve? Is there anything specific that is currently preventing this from happening? How can this be overcome?<br><br>what would be required to help develop policy to organise this? (probe – what data is needed, that research can contribute towards)
What about at a policy level -local or national government - is there anything you can identify that could help improve menstruation for your clients who are living in deprived circumstances?	What would this involve?
