# Supplementary material for: Period poverty: The perceptions and experiences of impoverished women living in an inner-city area of Northwest England
Source: PLoS One. 2022 Jul 14;17(7):e0269341. doi: 10.1371/journal.pone.0269341 (PMC9282460; doi:10.1371/journal.pone.0269341)
Supplement: S4 File — (PDF) [file pone.0269341.s004.pdf]

Coding Framework

Themes	Codes	Example Quotes	Comments
Thoughts on menstruation when asked general points	Negative	‘I hate everything about it but I don’t wanna not have it cos obviously then I…” “my actual period, I don’t have an issue, it’s before hand, all that emotional stuff and then I know that im due on with how I am in my self , I always go the same” “it just affects like being in work or just at home emotional, physically, sometimes, do you know what I mean, like the pain just gotta get on with it do you know what I mean and then like you always get someone with a smart arse comment like “are you due on ?” “I feel horrible, like greasy, spotty, eating all the time just everything about it, I just feel disgusting” but you’ve gotta get up and do it you cant have time off work or still got kids to look after, still got a house to run and still got shopping that needs doing” “I think i have quite heavy periods and a lot of the time ill go out and ill make sure im home in an hour or two cos if I have to change when im out it can be really messy because if you bleed really heavily and stuff, it restricts where im going and what I do when im bleeding” “it’s daunting having your period, it’s not a nice time of the month is it. It’s just”-M “when you say it’s daunting?”“feel washed out and anxious and irritable and frustrated” “when you know it’s happening you want it to just hurry up and happen” “definitely got a bit of a shame of it, like even the way on adverts they all use blue liquid, like why use blue liquid ? we all know what blood looks like -it just adds like that little bit of shame to it that its dirty and its wrong” “something I hate about being a woman” “something us women just have to endure unfortunately” “get on with it” “shouldn’t let it stop you doing anything” “I know [menstruation] means pain and it means discomfort for a good few days and…it’s just something us women have to endure unfortunately. It’s something I hate about being a woman, having to go through a period every month. You know, I once passed out with the pain it was so bad, I had to be sent home from work.” “Erm usually when I think im gonna start my period, I have to be well prepared with tampons and I know it means pain and it means erm discomfort for a good few days and its just something us women have to endure unfortunately. Its something I hate about being a woman, having to go through a period every month. You know, I once passed out with the pain it was so bad, I had to be sent home from work” it really disrupting your life, it has done, with the pain and the erm financial side of it, and depression and energy as well, just sap your energy as well but you sort of carry on. “no they’re not seen as healthy part,not seen as a normal process you know erm as I say its just something the women go through, they get on with it, keep it to themselves, its private and then there is a bit of proudness as well comes into it, cos it is a personal private thing er but yeah I think its just not freely open or freely spoken about, it’s just not out there, its still kind of like very old fashioned and I think that’s put a stigma over there I still think that that is affecting women now”	○ Most women<br>○ The word “embarrassed” used a lot<br>○ Dirty – feeling and blood<br>○ “just get on with it” mentality mentioned in all 3 FGD<br>○ Restrictions – don’t have sex mentioned few times. Swimming or activities where cant change.

		"but when I'm off the pill I get really bad pains and heavy periods"M "ok and how long would they last, your periods?"P "probably about 5 days and the period pain would be about 2 days. 2, 3 days. A day of that I'll be completely not able to do anything""in bed with a hot water bottle or a bath or painkillers yeah" M "yeah, so its -"yeah, completely debilitating"	
	Physical Sx	"i get really really really heavy ones so ive got to use a tampon and 2 pads on aswell and sometimes …"<br>"yeah so I take contraceptive pill now, mainly because of my pain, and it helps me manage my period pain"<br>"i get quite bad pain yeah, but I just take erm cocodamol or nurofen or paracetamol "<br>i used to take feminax and the only thing that was good about them was that it stopped me bleeding for a few hours, it didn't take the pain away but it slowed the bleeding a lot"<br>P9-i cant do anything when I'm on my period<br>P-all you want to do is lie down<br>it kills me. Sometimes of a morning, im like cant get out of bed cos the pains that bad<br>P-you don't get sick days as a mum<br>P-so you've gotta just get on with it<br>ive had periods where the pains so bad it comes in contractions, ive had to take time off work because I can't handle the pain but the majority of the time its just yeah im in pain but you've gotta crack on<br>'Achy, sleepy dragging'<br>"constant bleeding"<br>"Now, I think using drugs has been a part of self-medicating with the pain ive suffered. Cos I would, do you know if I'm having a period, im having medication from my clinic. I'd use the heroine over medication im given from the clinic cos it'd just melt away the pain."<br>"and ive had excruciating pain, I remember when I got really down to like 1ml on Subutex, I used to get excruciating pains and I remember this feminex and then I realise its got a bit of an opiate in it then I thought no wonder of course it wont work. You know because it blocks it a bit, the buprenorphine so erm you'd take like heroine and it'd be like stronger than the 1ml so it'd like you know, would be like override that then, take the pain away. It'd be nice that when the pain stops and then just feel like when I were a kid again"<br>so with the pain you experience on your periods, on a scale of 1 to 10 how bad would you say it is for you- id say it as a 10, definitely"<br>"I try not to go out as much if im really heavy."<br>"Erm, me mood changes when I know I'm coming on like I'll start eating more, I'll start lazing around more. I'm usually an active person. Erm I'll know when the pain starts and usually the day after that I'll come on.<br>because not just, sometimes they're embarrassed about being on periods and don't really want to talk about it, its personal, private"<br>"I think I have quite heavy periods and a lot of the time I'll go out and I'll make sure I'm home in an hour or two cos if I have to change when I'm out it can be really messy because if you bleed really heavily and stuff, it restricts where I'm going and what I do when I'm bleeding"	○ Leaking, flooding<br>○ Pain
	Psychological Sx	"feel washed out and anxious and irritable and frustrated"<br>"I just emotion and just, like cos I always have like dead heavy ones anyway, do you know what I mean and it was a nightmare, it just affects like being in work or just at home emotional, physically, sometimes, do you	○ PMT brought up without prompting

		know what I mean, like the pain just gotta get on with it do you know what I mean and then like you always get someone with a smart arse comment like "are you due on ?"<br>"I'm like when I'm due on, If I wasn't on anti-depressants, I have a really bad time"M "yep" -<br>"emotionally. I'm crying at the drop of a hat and stuff like that but erm but being on antidepressants has helped but, if I came off them, I would dread coming on every month."<br>"...Im crying at the drop of a hat and stuff like that but erm but being on antidepressants has helped but, if I came off them I would dread coming on every month"<br>"my actual period, I don't have an issue, it's beforehand, all that emotional stuff and then I know that im due on with how I am in my self, I always go the same"<br>I can feel like a horrible depression comes over me, its crushing and its scary sometimes and I do suffer with depression, but I do suffer with depression and its intensified with the PMT just before my period but ive got to recognise it over the years"<br>"it's daunting having your period, it's not a nice time of the month is it. It's just"M "when you say it's daunting"-<br>"feel washed out and anxious and irritable and frustrated"- "when you know it's happening you want it to just hurry up and happen"<br>"We can have women, even myself im having a really bad week, I have to remind myself, you're due on that's why your irritable, that's why you're ratty and I've kinda, we have these women with mental health problems who are struggling as it is, and then they're in absolute tears for a couple of days"	
	Positive	"it clears your system out doesn't it, gets you ready for your next cycle and stuff like that"<br>"I hate it, but then at this time in my life I think like umm yeah im glad ive got it because it means that im …"<br>"theres still potential ? fertile or whatever the word is" (mumbles)<br>*"for me, it's like I think that's my body, my healthiness coming back cos I don't have periods for like 3 years then I get like one out the blue then, sounds bad like but when I go to jail and I get my body healthy again, it's the day I get out of jail I end up coming on my period, so for me it's like my body's way of being healthy again its coming back you know what I mean"*	○ Mentioned once without prompting about periods = fertility for one woman<br>○ One lady – sign of healthiness
Products	Used / preference	"how can I explain it, just feel dirty using them, I don't think they're clean at all (pads)"<br>"my mum got erm toxic shock syndrome off tampons, so I've always like steered away from them because I just thought ill just use towels in case that happens to me"<br>"what I do is, I try to get whatevers available and then try to get the super plus. Now you can get super, if I can get my hands on super plus tampons im like yeah, do you know stocking up on them. Id get super or super plus and it doesn't have to be branded, cos they're too expensive. I would like them, yeah they're more comfortable they do seem a bit better for some reason than er the cheaper brands. Id get, id always get pads aswell so it all adds up doesn't it?".<br>"yeah, uncomfortable a lot of the time, I just put up with it. Especially the pads, the tampons aren't a problem but sometimes the cheap ones, they can like I don't know, work their way down for some reason I don't know whether it's the shape or what. Ive flushed them down the toilet cos they've been no good. Even though they are a decent size"<br>"we do,we do. we get regular donations, erm im not 100% sure who they're from now erm but we do get one a month some donations. The only problem is its very few tampax or tampons, its mostly pads". "now, A lot of women don't like wearing pads yeah they prefer wearing tampax, tampons, erm so we have got loads of donations …? donations.. sometimes erm if we know there is a resident, like we've got a resident with endometriosis so we'll regularly we'll say to her "do you need any pads?" er stuff like that again the residents	○ Tampons or pads<br>○ When asked about brand vs non brand all women use own brand<br>○ Contraception to manage/reduce periods

		know we have them but sometimes they're not that for coming in asking for them" I" why do you think that is?"- "er out of embarrassment"<br>"but we do have a good stock but we've got loads of stock because the women prefer tampax not er pads yeah sometimes they just feel it's a little bit cleaner to wear tampax er and its just not as problematic, you know sometimes you're wearing a pad, a pad and a little bit conscious of is it leaking and all that type of stuff that comes with it"	
	Cost	"-i use store own brands tampons M-sorry you use?-"you know like asda own brand-they're expensive (mumbled)"<br>"but if you're struggling to buy towels or tampons. I cant, the ones ive looked into was like £30 or something like that, that's a lot in one lump sum if you're struggling to pay for any other kind of sanitary products"<br>"sorry ill just say, if I buy like soap powder anyways but I have to do loads more washing cos I bleed so heavily so I leak when I'm in bed I have to wash loads of pyjamas, loads of knickers, so even that like adds to the cost i buy asdas own for 77pence, box of tampax, asdas own. If I bought a normal box of tampax, we're talking £3.49 or something like that for 16"<br>"cos ive got 2 daughters aswell so I could spend like £10 a month on sanitary stuff"<br>"you only get a set amount of benefits so when you get your benefit it might not be your menstrual week, do you know what I mean, and obviolsy that doesn't come into your head. So when you've got no money and it does happen or sometimes, you can, I used to have two periods a month, quite often, do you know what I mean, so what are you supposed to do then ?"<br>"oh it costs me a fortune, do you know, im like, I can easy spend £10 a day, and I know that sounds bizarre. Buying products, going through box after box, ive even had to, and I hate to say this, but because ive had no money and all the toilet rolls ive spent and blooming one thing with the washing and stuff, washing your bedding, ive had to erm steal tampons cos ive run out and I cant afford any more do you know cos they're like £2, £3 some packs, you've gone into some shops and specially if you've had an accident and tesco is closed and cant get up there. You haven't a pay these corner shop prices which are, some of them are ridiculous you know them little lillets."<br>"Yeh erm, ive always got to try and prepare and keep some in but its expense, it is, which I could really do without. And its like we get a bit exploited don't we cos they know we need them, so they can charge what they want, oh women need them just charge"<br>"yeah and I do try to get those that are at a discount price because you know I cant afford tampax. I mean if I go in a shop and they haven't got anything else, I will have to buy them and im like urgh ive had to pay an extra £1, £1.20 something"<br>"I think they'd be much cheaper if men had periods (laughs)probably free don't you think"<br>"Oh my clothing yeah, clothings got damaged. Ive always got knickers soaking in a bucket, I have now (laughs)."<br>"if it was the choice between five pound on (menstrual) products and five pound on gas and electricity,it's gonna be the gas and electricity."<br>"Erm, I think they are quite affordable to be fair. It depends what make it is though cos the proper thin ones they don't work at all and you are paying all this of money for them".<br>"Yeah. I have had to struggle to find money to get them 'cause mine can be very heavy"<br>"well I'll just give you an example … you know as I said we're struggling a little bit, and when I realized - cos me app it gives me, it tells me oh you're due on your period in a couple of days and I thought 'oh, god what have I got in?' you know what I mean because we've got no money at the minute. And luckily I did have about six [tampon brand] left so I thought you know what - and I know this sounds terrible - but with me being at home and that I'll just have to like you know budget the ones I've got and just make them last and if I've got to use tissue I'll use tissue in between."	○ When asked about buying products- negative "hate buying"<br>○ Often "expensive" is mentioned without prompt<br>○ Not budgeted for – Universal credit or benefits mentioned "living hand to mouth"<br>○ When asked about keeping clean – soap powder brought up as expensive

	Improvised products /managing when cant afford	“but years ago when I was (mumbles) 9 times out of 10 ive done it, ive gone in and shoplifted, I really have or ive had to rely on going around a friends house and said can I borrow me some quite a few times”<br>“I’ve used tissue a few times -P-yeah I have - P- got the toilet roll, fold it here, fold it here, fold it here -P-its quite common, like when if my daughter’s ran out”<br>“yeah, yeah, definitely. I have yeah, do you know wearing it when I shouldn’t have worn it and getting the thickest one I could find and even putting toilet paper up there do you know to help it 1 inside?2 yeah and cotton wool 2 definitely to make it last longer cos I cant afford anymore 1 has that happened to you a few times or ? 2 lots of times, id say I’ve run out of tampons and I’ve had to try and make my own with cotton wool, lots of toilet paper, going through toilet paper, robbing toilet paper from food shops and stuff, I wouldn’t take the roll like some people, wouldn’t do that but going in places and getting toilet roll cos im running out, its awful. Like ive just had a period now and ive gone through a load of toilet roll”<br>”you can’t afford to go and buy like 7 packets, can’t afford to just go pay 3,4 pound odd a packet do you know what I mean, that’d be most of my money, do you know what I mean, and I’ll be honest, [tampon brand] I robbed cause they’re smaller”<br>“I robbed them, because I can’t afford to pay for them” M“how do you feel about having to do that?”- “get caught, the embarrassment of getting caught and you’re taking that risk of reoffending and going to jail “what are you in for?” “a box of tampons” (laughs) M“do you think its common that people have to do that” - “yeah” - “yeah it will be. If they’re honest, yeah, especially in this environment”<br>“I think (mumbles) when they’re in a longer time than usual, do you know what I mean, because say if I’m here I’d change them more often but if you’re on the street and you don’t have that access you do try and have them on longer and that’s when they will start chafing”<br>“yes. Because I’ve run out of money”... “and I’ve been so heavy and its usually fallen on a week where I don’t have much money” … “and I have had to go into a shop and rob tampons to get buy.” … “2 yeah I’ve had to do that. Or sit on a towel, see me sitting on a towel and not able to go out the house”<br>“erm did you ever erm exchange sex for money so you could buy menstrual products?“yeah, yeah definitely” … “when I were working, cos I was on drugs, definitely. Yeah”<br>“I’ve had to use socks before today”-“I was just about to say, thank god you said that” “I have done”- “I have done” “I’d rather, you know, use something clean than nothing at all, you know what I’m saying, I’ll do what I’ve got to do, know what I’m saying”- “don’t want it leaking into your pants and then it’s on show”<br>“sometimes yeah. What id do is, put loads of toilet paper up there so I could still work” ... “ because I, or a sponge, put a sponge up” “are you with me, so it’s like always flaming on and I was trying to work for money so I’ve seen me do that. Had to block myself up to do the odd job yeah (laughs)”<br>“ I have to use toilet roll down there. It’s very embarrassing if like - you know. I don’t like using toilet roll. but then they must kinda because we don’t get that many asking for the products aswell, so they must be sorting themselves out in a way, how they go about doing it, I don’t know, you know”<br>“F you’re not sure, so potentially if they’re not getting, if they’re not spending the money on products and they’re not getting them through the hostel itself potentially I guess they could be getting them through friends or they could be using other things, yeah using other things or robbing them yeah, because you know, they don’t budget for sanitary products “<br>“and even though, like a lot of the, even the chaotic women who, you know, their priorities are completely different, there’s a lot of erm proudness that goes with it as well”	○ All mention tissue/toilet roll as improvised product<br>○ Socks, pillow case used<br>○ Shop lifting “robbing”– when asked if common “yes” replied.

		"get caught, the embarrassment of getting caught and you're taking that risk of reoffending and going to jail "what are you in for?" "a box of tampons" (laughs)M -"do you think its common that people have to do that" - "yeah" - "yeah it will be. If they're honest, yeah, especially in this environment"	
	Reusable products – when asked	"so whys nothing advertised or no information on them nothing like that"<br>" the price is gonna deter people. Like I say, products, some people cant afford them. So they're advertising this at £20-30<br>"think they'd be offering them out at family planning clinics"<br>"in terms of say plastic, yeah it's a good idea, but I don't wanna sound disgusting ? washing it and all but I don't use that many products cos its only probably a day or something, and I cant see myself taking it out and washing it"<br>"I think it's the fear"<br>"think you'd be wasting as much water as you are plastic and stuff like that, do you know what I mean"<br>-"ive seen those reusable sanitary towels that you wash and apparently they're all organic and they've made with some special organic stuff that draws the blood from you so it doesn't cause pain and relieves stomach cramps or something"<br>"i cant see how a cloth is absorb (mumbles) and you get things like clots and nah just the fact of thinking im washing something knowing what happens in periods and that and then im going to be wearing this again, I just wouldn't do it"<br>P-im not personally disgusted by it and I think it's a good idea and im tampons and that have bleach in them so if you're using tampons you're inserting all those chemicals into your body. So like Menstrual cups are a good idea but it's the practicality of them, like public toilets, if you have to take them out and then wash it, most public toilets, you'd gotta go into the cubicle, empty it, come out of the cubicle, use the sink to wash it, again its not very private because you're in the open area of the toilets -P-disgusting -P-its not massively hygienic, and then you'd have to go back in to put it back in P-urgh- P-so I don't think it's very practical with the public toielts that we've got"<br>"you know those cup things, something about menstrual cups, I wish id known about them"<br>"I haven't heard of no other products than towels".	○ Cups – not heard of<br>○ Negative : price too high and "disgusting" opinion common<br>○ Positive for waste reduction<br>○ Pads One lady had heard of<br>– negative: issue of washing them. Old fashioned
Public changing facilities – when asked		"about a time when there were plenty of public toilets around but now there is none. If the girls wanted to go to the toilet, which I don't get anymore but other people they're in a rush and need to get to the toilet"<br>"no I think there's a lot less access to public toilets now, there used to be erm just buildings that were public toilets, wasn't there, but there aren't any more"<br>"theres people with weak bladders and things like that you need to use the toilet and you go into tescos or wherever yous go to, and see a queue there and think oh no im not gonna make it. Terrible isn't it"<br>"or if you go in a shop or a pub or a café or something they're like no its for customers only and.."<br>"i used to change mine before I went out then if I was like shopping in the town centre with my mum, id only go and change my sanitary pad in the sexual health clinic cos they'd let me go to the toilet for free in there and provided free products as well"	○ Mixed opinion<br>○ Negative<br>– pay to use<br>– for customers only<br>○ "Less access now to public facilities"<br>○ Homeless women no WASH facilities

		"sometimes that's been difficult, had to go in a bush, you know before today. If I hadn't have been able to get to a shop and all the toilets are locked. Do you know, you've said have you gone in a toilet, erm erm that erm subway and other place, I don't know, you cant go in unless you were eating, don't want people going in, you know, go in people aren't allowed to use, toilets aren't working they'd say "Toilets not working" I just think they didn't want me in. you know to go and change"<br>"yeah yeah oh yeah obviously they think she's going in to do drugs or something or do you know rob toilet roll or something yeah erm yeah probably yeah. Maybe that's why I've been denied access"<br>yeah they all have sani bins in their room but to be honest a lot of them don't use them. They just put them in a little bag and take the rubbish out"<br>"yeah you have to pay like 20p or 50p or something erm for the toilets with the automated doors and all that".<br>P6 "yeah the ones in the train stations they are about 40p or something now aren't they. Have to pay to go to the toilet, it's ridiculous!" -"yeah that's ridiculous, especially for us women, you know you're not really having the choice are you, if you've gotta go you've gotta go"<br>'its hrrible living on the street …it's hard enough to get washed and you can't in the shop and sometimes I've into the shop and rob them [products], being honest … they've been that bad'<br>'and you're using tissue and wipes, baby wipes, to wipe yourself down below …because you haven't got access to somewhere to have a shower or a bath'<br>'and its not nice cos its not even that, people know you are homeless in town so they don't even want you going into the toilet'	○ Some women didn't find being out a problem<br>○ Some tried to only change at home<br>○ Disposal of products wasn't a problem when asked
Solutions – when asked	Free for all / some women	"they should make a government scheme where they issue them as a one off issue it out a one off scheme"<br>-they should be free, we didn't chose to have periods, more understanding"<br>"I think they'd be much cheaper if men had periods…probably free don't you think"<br>"it should be spoke about more often cos men tend to not speak about it and shy do you know what I mean but if men had an understanding as much as women do that'd be half the battle, to be honest. they don't have to go through it"<br>"I think that as well the way you think you have to go and buy sanitary towels, tampons and stuff like this yet you can go to the sexual health clinic and get condoms for free. Do you know what I mean, you chose to have sex, you don't choose to have periods do you know what I'm saying so I don't get why…why you have to pay for them"<br>"more facilities, yeah not, they're very very scarce if you go into public bathrooms"<br>"I think they'd be much cheaper if men had periods (laughs)probably free don't you think"<br>"I would, I'd like to see them more freely available at erm erm a price that people can afford. Affordable and even maybe free for women do you know that are struggling, that are on benefits. Go to the doctor maybe. Especially If you're having heavy periods, get them on prescription"<br>"condoms are free"<br>"it's not a luxury to prevent having blood running down your legs is it. That's not a luxurious thing, that's just practicality"<br>"I just think they should hand them out to you wherever you are".<br>"they hand condoms out don't they so what's the difference?"	○ General view point of all women<br>○ All women vs those who need. "care of the chemist" "on prescription"<br>○ "condoms are free"<br>○ Free in public places

	Education	“you know, so I think they education and awareness of a healthy vagina full stop. That needs to be spoke about a bit more as well and a bit more erm I think maybe start off at the educational level, from school, and I think its important that boys as well as girls”<br>“so its open you know girls aren’t gonna talk about it infront of lads in school but I think it should be taught. You know that personal education or PSE or something like that”<br>“it follows plus I think girls are more likely to have a little talk amongst themselves but I think because of that influence when you’re a teenager and you like a lad and stuff like that, lads like grossed out by it urghh and all that so I think that again impacts girls “<br>“and then it follows them further on into adulthood then the lads are no better educated so they’re still growing up with wives and children, I don’t wanna know I don’t wanna know, you know what I mean. So I think education on a whole, start off in schools and maybe in address in science, they touch slightly on the reproductive systems but they don’t talk properly about body functions, and I think educate the kids in school first, you know, destigmatise the take all that away”<br>“more of a discussions, educating the women, telling them where they can go, erm you know, needs to be opened up a bit more and something more for women. A lot of them you can take to them about stuff they don’t even wanna go to the doctor and especially if the doctors a male”<br>“I think the important thing and why I said I want to talk a little bit about this today is because from a man’s perspective it’s not as straightforward as it would be for one of my colleagues. I have 2 women colleagues in the management team, they don’t seem to have a problem with it but that’s rightfully so, but we need to be aware that maybe 20% of the volunteers are male and for them it’s not always straightforward. Erm it’s not easy, and … I think that’s something we need to be aware of and I think us men need to know it’s something we need to make sure it’s a need that’s met and are not convinced we know all the answers to”<br>it’s having an awareness. Think the staff need to put out the support side of it for the well-being you know, asking them, “are your periods regular?” reminding them we’ve got products, asking them if they need products er you know getting a bit of a feel to whether people are going through the menopause, because that brings other things with it… the changes, the hot flushes, stuff that we should be putting in and supporting them around and maybe you know, advising them on it, accessing their GP, talk about HRT or other alternatives… and I think just because of the current feel of it all, and it’s all kind of behind doors, its forgot about, even with staff now sitting here talking about it, it’s all coming out to light its thinking that should be one of our starting blocks”	○ In Schools?<br>○ Men and boys included
	De stigmatise / ask / talk openly	“yeah that’s modern day but I think theres still very old fashioned values are tied to women and periods and I think that has a massive influence over it and I think whilst thinking and talking about stuff im thinking, I think really us staff should on a daily basis be asking the girls do you need pads, do you need- we do it occasionally but it’s not every day practice. I think to kind of break that barrier because its so quiet and secretive the women just kinda get on with it “<br>“but I think erm staff need to kinda talk about it more you know, and maybe ask people cos id spoken to [name] and she said about her periods but then its more cos she hadn’t had in [number] months. Do you know what I mean, so its having an awareness, Think the staff need to put out the support side of it for the wellbeing you know, asking them, are your periods regular reminding them we’ve got products, asking them if they need products er you know getting a bit of a feel to whether people are going through the menopause, because that	○

		brings other things with itand I think just because of the current feel of it all, and its all kind of behind doors, its forgot about, even with staff now sitting here talking about it, its all coming out to light its thinking that should be one of our starting blocks"<br>"and I think it needs to be more open and talked about to break them stigmas"<br>"we work in a women's service, … for women, about women but I think it's just because of the whole area of it, it's [menstruation] not on our radar its kinda just pushed to one side"<br>'the staff need to put out the support side of it for the well-being you know, asking them, are your periods regular, reminding them we've got products, asking them if they need products".	
