# Supplementary material for: Period poverty: The perceptions and experiences of impoverished women living in an inner-city area of Northwest England
Source: PLoS One. 2022 Jul 14;17(7):e0269341. doi: 10.1371/journal.pone.0269341 (PMC9282460; doi:10.1371/journal.pone.0269341)
Supplement: Attachment — Submitted filename: Response to Reviewers 5May.docx [file pone.0269341.s006.docx]

Dear Alison Parker

Thank you very much for reviewing our paper, ‘*Period Poverty: The perceptions and experiences of impoverished women living an inner-city area of Northwest England’*. Please note this was previously entitled “*it’s not a luxury to prevent having blood running down your legs”. The experiences and perceptions of period poverty among impoverished women in an inner-city area of Northwest England*.

We read the reviewers comments with interest and are grateful for their suggestions to strengthen our paper. We hope our amendments meet with their approval. These are outlined below, following details of our alterations to meet the journal submission requirements.

We hope this response meets with your and the reviewers approval.

Best wishes,

Linda Mason

Senior Research Fellow

Liverpool School of Tropical Medicine, Pembroke Place, L3 5QA, Liverpool,

**Journal Requirements:**

**When submitting your revision, we need you to address these additional requirements.**

**Please ensure that your manuscript meets PLOS ONE's style requirements, including those for file naming.**

- We have named our files in accordance with the guidance outlined below. A rebuttal letter is named 'Response to Reviewers 5May'. The marked up manuscript copy is labelled 'Revised Manuscript with Track Changes 5May'. The unmarked version without tracked changes is uploaded as 'Manuscript 5May'. For the supplementary files now included we have followed the guidance and labelled them Supporting Information 1, Supporting Information 2

**Please review your reference list to ensure that it is complete and correct.**

- Additional references have been included as per the reviewers’ suggestions. These are:

3.	Hennegan J, Winkler IT, Bobel C, Keiser D, Hampton J, Larsson G, et al. Menstrual health: a definition for policy, practice, and research. Sexual & Reproductive Health Matters. 2021;29(1):31-8. doi: 10.1080/26410397.2021.1911618.

6.	The Scottish Parliament. Period Poverty Bill. 2021.

18.	Plan International. Periods In A Pandemic: Menstrual Hygiene Management In The Time Of COVID-19. 2020.

19.	Plan International UK. The State of Girls’ Rights in the UK – Early insights into the impact of the coronavirus pandemic on girls. 2020 May.

22.	Bobel C, Fahs B. From Bloodless Respectability to Radical Menstrual Embodiment: Shifting Menstrual Politics from Private to Public. Signs: Journal of Women in Culture & Society. 2020;45(4):955-83.

34.	Crawford BJ, Spivack C. TAMPON TAXES, DISCRIMINATION, AND HUMAN RIGHTS. Wisconsin Law Review. 2017;2017(3):491-549. PubMed PMID: 123889808.

- No reference has been retracted.

**We note that you have provided funding information that is not currently declared in your Funding Statement. However, funding information should not appear in the Acknowledgments section or other areas of your manuscript. Please include your amended statements within your cover letter; we will change the online submission form on your behalf.**

- We have removed the information from the acknowledgment section that was pertaining to funding. Please include the amended statement below on the online submission.

*Preparation of this paper was supported by the UKRI ‘Enhancing place-based partnerships in public engagement’ grant. The reference number for this grant is UKRI/EPPE19/13*

*The grant holder was PPH. The funders of this study had no role in study design, data collection and analysis, decision to publish, or preparation of the manuscript.*

**In your Data Availability statement, you have not specified where the minimal data set underlying the results described in your manuscript can be found.**

**Upon re-submitting your revised manuscript, please upload your study’s minimal underlying data set as either Supporting Information files or to a stable, public repository and include the relevant URLs, DOIs, or accession numbers within your revised cover letter.**

- Please accept our apologies for this erroneous statement. All data are now available from the UK Data Service Reshare. https://reshare.ukdataservice.ac.uk/855592/ (However they have placed under permission only access. Contact authors Penelope Phillips-Howard or Linda Mason)
- We request that the Data Availability statement is amended to read ‘All data are now available from the UK Data Service Reshare, under permission only access. https://reshare.ukdataservice.ac.uk/855592/’
- We have included, as requested by Reviewer 1, two additional files with supporting information. SI1 includes the interview schedules. SI2 includes a codebook.
- **Reviewer #1:**

**This is a well-evidenced report of a study investigating the realities of 'period poverty' for women in a city of England. the use of illustrative quotes is sound. It's a shame that the sample size is relatively small due to COVID-19, but I recommend publication as there is so little existing information on these experiences. I do however think that there are a few improvements that could be made to the manuscript in terms of being clearer, and providing some extra details in the Supporting Information; I have highlighted and noted these on a pdf of the manuscript. I don't think these are overly burdensome and I don't think the paper should be accepted until they have been addressed. They are all related to presenting the study more clearly; I don't have any issues with the study itself.**

- Thank you to reviewer 1 for the helpful feedback. We hope that our amendments as suggested meet your approval. These are as follows:
- We have amended as per suggested all of the grammatical corrections.

Introduction

- We are cognizant that the term ‘menstrual health’ has been suggested by the Terminology Action Group of the Global Menstrual Collective in the published paper Menstrual health: a definition for policy, practice, and research by Hennegan et al (2021). Indeed, we gave due consideration to amending the definition used in our study. However, on balance we noted that the womens’ experiences heavily emphasised the ‘hygiene’ aspect and so in this instance we felt using the original definition of menstrual health and hygiene, was the most appropriate under the circumstances for this particular paper. We note this is not only currently used in many academic references, but is also the terminology used by the menstrual health and hygiene (MHH) group funded through Bill and Melinda Gates Foundation to develop MHH indicators for national governments.
- The suggestion to amend the sentence referring to societies need to change now reads *However, stigma and societies’ attitudes also need to change in parallel, to educate, support and empower menstruators, reducing period shame along with dangerous traditions [5]. Whilst the present study focuses on period poverty, it is recognized this is just one facet of MH that needs addressing.*
- The comment referring to food insecurity has been amended to clarify that the high school study did not mention this specific aspect. This now reads: *Both studies found a significant association between period poverty and food insecurity. Period poverty was also documented in a small study of 58 high school students [9] with almost half of the students unable to afford products during at least one menses in the school year, whilst 12% were unable to afford them ‘most’ months* [9]
- We have included a supporting statement to indicate what the authors classed as ‘vulnerable’. *A phenomenological study interviewed 40 ‘vulnerable’ women in the UK i.e women accessing homeless shelters, drug support groups, day centers, and foodbanks.*
- The paragraph that included reference to close confinement and privacy has been rewritten in line with reviewer 2’s suggestion. Please see below.

Methods

- We have included the FGD and IDI topic guides as Supporting Information 1.
- We have omitted reference to ‘selected days’ and clarified which site the recruitment took place in the participant recruitment section. ‘*For IDIs with women, researchers attended the Site 3 foodbank and recruited women opportunistically after handing out fliers outlining the project.*
- The codebook example has been included as Supporting Information 2.

### The statement referring to the consent process has been clarified and now reads ‘*The research team obtained informed consent from all participants prior to each IDI / FGD. As some of the women may have been illiterate, they were reminded verbally of the purpose of the study, what participation would involve and given the opportunity to ask further questions. They were asked to provide a ‘x’ to denote signature as a sign of consent. Staff were similarly reminded the same information but provided their written signature to denote consent’.*

Results

- We have amended the coding as suggested and have included a ‘W’ prior to the womens’ IDI, and continued with the prefix of ‘staff’ prior to these interviews. A sentence to denote this has been included ‘*Womens’ interviews were assigned ‘W’ before ‘IDI’ whilst staff interviews denoted ‘staff’*
- The sentence referring to coping with menstruation now reads ‘*Some participants described changing their daily routine to cope with their menstrual flow*’
- We have included a quote to illustrate the suggestions made by participants that men and boys need to be taught about menstrual health. *‘lads like grossed out by it urghh and all that ………so I think that again impacts girls ……and then it follows them further on into adulthood then the lads are no better educated so they’re still growing up with wives and children, ‘I don’t wanna know I don’t wanna know’, you know what I mean. So I think education on a whole, start off in schools and maybe in address in science, they touch slightly on the reproductive systems but they don’t talk properly about body functions, and I think educate the kids in school first, you know, destigmatise, take all that away’* (Staff IDI03 Supported accommodation)
- We have inserted the following comment in relation to the question ‘do you think there is an issue with how the availability of free products is being communicated?’ ‘*Despite sanitary products being made available in schools and also on an ad hoc basis in offices, public houses, shopping centres etc, this was not acknowledged by our participants. We conjecture this may be because the availability is not extensive, nor advertised widely in the circles that impoverished women access, unlike provision of condoms’.*
- Regarding the issue of male decision making we have inserted the following to clarify this is our conjecture only. *‘Furthermore, we speculate that if the men making these decisions in government roles had a better context and understanding for women’s health, such issues may not exist’.*
- We have inserted a reference to illustrate our suggestion that in the US sanitary products may be considered a luxury. *‘Similarly, in the US, sales tax paid on purchase of menstrual products exists because most states consider these products a luxury or, at the least, not a necessity, likely stemming from the historical view that tampons were considered as cosmetics [34]’*.
- Our introduction now clarifies the situation in Scotland and includes a reference to the parliamentary bill introducing this. ‘*In 2020 Scotland became the first country to make period products free for all which meant local authorities became legally obliged to provide free menstrual items to anyone that needs them [6]’.*

Discussion

- We erroneously used the name Branigan and have corrected to read ‘Barrington’.
- We have not included specific mention to power differentials as we are of the opinion that this is reflected within the paragraph which mentions are awareness of the variations in socio-economic circumstances (wealth, occupation, education), and how we used a reflexive stance as we were mindful of our positionality throughout both data collection, analysis and writing up of the paper. We hope this will suffice.
- We would like to thank reviewer 1 for the positive comments referring to specific points that we included.

**No Supporting Information has been provided as far as I can tell, can these please be made available to reviewers if this paper goes for another round of reviews (I understand this may be the system's, not the authors', fault)? I answered 'No' to Q3 above because I have not seen the data/there is no DOI provided in the manuscript, but the authors do state that "all data are fully available without restriction" and "All relevant data are within the manuscript and its Supporting Information files."**

- Please see the above statement within the journal submission requirements section.

**Reviewer #2:**

**I would suggest dramatically reducing the length of your paper's title. It is quite unwieldy. If you insist on using the quote, consider using it as a subtitle, not the primary title.**

- We have amended the title which now reads *‘Period Poverty: The perceptions and experiences of impoverished women living an inner-city area of Northwest England’*. We hope that this meets with reviewer 2 approval.

**Also you need at least a paragraph explaining your decision (a perfectly valid one) to refer to "women and girls" instead of "individuals who menstruate," "menstruators," or "women, girls and other individuals who menstruate."**

- We feel this was a valid point and thank the reviewer for picking up on this. We have amended the narrative to read ‘menstruators’ when referring in general. Where we are using specific examples as per the literature, we have used the same terms as adopted by the authors. We have included the following in the introduction *(In our paper we interweave terms pertaining to women, girls and menstruators as appropriate and according to sources of information we cite)*

**There are some relevant sources the authors have overlooked:**

**(1) PLAN INTERNATIONAL, PERIODS IN A PANDEMIC: MENSTRUAL HYGIENE MANAGEMENT IN THE TIME OF COVID-19 (2020)**

**(2) Chris Bobel & Breanne Fahs, From Bloodless Respectability to Radical Menstrual Embodiment: Shifting Menstrual Politics from Private to Public, 45 SIGNS 955 (2020) (for discussion of "menstruators" vs. "women and girls")

(3) Anne Siebert Kuhlman, Unmet Menstrual Hygiene Needs Among Low-Income Women doi: 10.1097/AOG.0000000000003060

(4) Crawford & Waldman, Period Poverty in a Pandemic,** [**https://papers.ssrn.com/sol3/papers.cfm?abstract_id=3692802**](https://protect-eu.mimecast.com/s/DcRFCgnOESlB26QhkxSMD?domain=papers.ssrn.com)

- All of these references have now been included, and are illustrated below (with further references also inserted). We believe this has strengthened the introduction and discussion sections. Please note however, we agree that the work by Kuhlman is important to this topic and had already included the reference suggested (Reference 7).

*The current COVID-19 pandemic has ‘amplified’ the marginalisation and vulnerability of menstruators worldwide, according to Crawford and Waldman [17]. They identified 4 key areas of concern; access to affordable products, maintenance of hygiene and sanitation, menstruation information and support, and privacy and dignity issues arising through restricted movement.*

*Similarly, Plan International [18] conducted a small survey of global WASH (Water, Sanitation and Hygiene) professionals who identified that difficulties already ongoing were made even more challenging through restricted access to products, WASH facilities, information, and increase in price of products. Such challenges appear not confined to menstruators in LMIC but also experienced amongst those living in the UK and other HIC. Plan Internationals’ UK survey [19] conducted during the pandemic found 11% of girls aged 14-21 could not afford period products and were improvising with makeshift products, whilst 22% of girls who could afford period products were struggling to access them mostly due to lack of availability in shops.*

**(5) Any mention of Scotland's adoption of national legislation providing free menstrual products**

- Thank you for this suggestion. We have included information on the Period Poverty Bill and discussed in more detail in the introduction. ‘*There has been some momentum to make menstrual products more accessible including efforts in HIC. Canada and Australia scrapped tampon tax in 2015 and 2018 respectively, with the UK joining in 2021. In 2020 Scotland became the first country to make period products free for all which meant local authorities became legally obliged to provide free menstrual items to anyone that needs them [6]). Whilst in the rest of the UK, advocacy campaigns and actions (e.g. Pink Protest, Red Box presentations to Parliament) have resulted in legislation to provide free menstrual products in UK primary and secondary schools’.*

**You might consider, although this is the weakest of my recommendations, engaging more robustly (in two or three more paragraphs) with the existing literature on period poverty. Some important scholarship has been overlooked.**

- As per the suggestions for additional references and mention of the situation in Scotland we have expanded the literature review and hope it meets with the reviewers approval. Please note this is not as per suggested in 2 or 3 more paragraphs, but rather woven in to the existing literature as we felt appropriate.
