# Supplementary material for: Period poverty: The perceptions and experiences of impoverished women living in an inner-city area of Northwest England
Source: PLoS One. 2022 Jul 14;17(7):e0269341. doi: 10.1371/journal.pone.0269341 (PMC9282460; doi:10.1371/journal.pone.0269341)
Supplement: Attachment — Submitted filename: Response to Reviewers 17May.docx [file pone.0269341.s008.docx]

Dear Alison Parker

Thank you very much for reviewing our paper, ‘*Period Poverty: The perceptions and experiences of impoverished women living an inner-city area of Northwest England’ again*.

We wish to think Reviewer 1 for taking the time to provide such meticulous feedback. It has been very helpful indeed and hopefully we have done justice to her efforts to improve our paper.

All grammatical suggestions have been addressed (we have not itemised in this response), and having been alerted to errors we have, in addition, incorporated suggestions from a proof-reader. There are two issues we provide detail on.

1) The suggestion to group the US studies versus other studies was attempted on an early draft for the very reasons outlined by Reviewer 1. However, in doing this the narrative became quite complicated. We reverted to a narrative that groups studies by method and findings, which does require mention of the geography of each study, but overall reads, in our opinion, more straightforwardly.

2) We understand the point made regarding our original response to the Bobel and Fahs article. On re-reading we now agree that Bobel and Fahs were arguing the need for more evidence before assertions are made. Our study helps in providing additional insight to the topic and we now have clarified this point. “*A dominant theme emerging from our study concerned affordability of menstrual products. We found our participants believed better access to products would have a significant influence on their lives. Bobel and Fahs [22] asserted that “It is troubling that activists assert the power of products to impact lives when the research base for these interventions, thus far, is thin” (p975). Our study has provided further evidence* *indicating the importance of product affordability for vulnerable women, which is likely to become even more critical within the UK given the current economic crisis”*

We apologise for the loss of some track changes in the previous version sent which we appreciate must have made this more difficult and time consuming to check our amendments. Unfortunately, we have been unable to highlight this last set of track changes in a different format, which would have made it easier to check and we thank you for your patience.

We hope this response meets with your and the reviewers approval.

Best wishes,

Linda Mason

Senior Research Fellow

Liverpool School of Tropical Medicine, Pembroke Place, L3 5QA, Liverpool,
